# Wild ginseng adventitious roots restore sleep homeostasis in sleep-deprived *Drosophila melanogaster*

**DOI:** 10.3389/fphar.2026.1907250

**Published:** 2026-10-02

**Authors:** Hong Chen, Xiangzhu Li, Hang Chi, Yongjun Zheng, Yingna Li, Lu Xing, Rui Ma, Liwei Sun, Xuenan Chen

**Affiliations:** 1 Shanghai Jiao Tong University, Shanghai, China; 2 Tonghua Herbal Biotechnology Co., Ltd., Tonghua/Jilin, China; 3 The Affiliated Hospital to Changchun University of Chinese Medicine, Changchun, China; 4 Key Laboratory of Active Substances and Biological Mechanisms of Ginseng Efficacy, Ministry of Education, Changchun University of Chinese Medicine, Changchun/Jilin, China

**Keywords:** cellular homeostasis, *Drosophila melanogaster*, ginseng adventitious roots, sleep deprivation, sleep regulation

## Abstract

**Introduction:**

Sleep deprivation is increasingly recognized as a major health concern and is associated with impaired physiological and neurological functions. *Panax ginseng* C. A. Mey. has long been used to improve sleep quality; however, the limited availability and long cultivation cycle of natural ginseng restrict its sustainable utilization. Wild ginseng adventitious roots (ADV), produced via plant tissue culture, provide a renewable source of bioactive ginsenosides, although their potential role in sleep regulation remains poorly understood.

**Methods:**

The sleep-promoting effects and safety of ADV were evaluated in a sleep deprivation-induced *Drosophila melanogaster* model. Sleep parameters, including total sleep duration, sleep latency, deep sleep, and sleep fragmentation, were assessed, together with addiction-like behavioral assays. Transcriptomic analysis was performed to investigate the underlying mechanisms. LC-MS/MS and bioactivity assays were conducted to identify and validate major active ginsenosides.

**Results:**

ADV significantly improved sleep behavior in sleep-deprived flies, increasing total sleep duration and deep sleep while reducing sleep latency and sleep fragmentation, without inducing addiction-like behaviors. Transcriptomic analysis revealed that ADV modulated multiple sleep-related biological processes, particularly stress response pathway, as evidenced by altered expression of *Hsp68, Hsp70Aa, Hsp70Ba, Hsp70Bb,* and *Hsp70Bbb*. Combined with LC-MS/MS identification and biological activity comparison, ginsenosides Rc and F2 were preliminarily identified as the principal active metabolites.

**Conclusion:**

In the *Drosophila melanogaster* sleep-deprivation model, ADV treatment is associated with improvements in sleep-related behavioural parameters alongside altered expression of conserved stress-response-related biological processes. These findings support ADV as a sustainable source of bioactive ginsenosides, though the current experimental design cannot establish a strictly sleep-specific or causal mechanistic relationship.

## 1 Introduction

Sleep deprivation (SD) has become increasingly prevalent due to modern lifestyle factors such as prolonged screen exposure, irregular work schedules, and extended working hours. Insufficient sleep is closely associated with cognitive impairment, emotional dysregulation, and increased risk of metabolic and neurological disorders (Dieter et al., 2019). Although pharmacological agents such as amphetamines, caffeine, and modafinil are commonly used to counteract sleep loss, their long-term application is limited by adverse effects and dependence liability (Tobias et al., 2018). Therefore, the identification of safe and effective sleep-regulating agents remains an important pharmacological challenge.

*Panax ginseng* C. A. Mey. (ginseng) contains diverse ginsenosides that exhibit a broad spectrum of neuropharmacological activities, including anti-fatigue, antidepressant, neuroprotective, and sleep-regulating effects (H F et al., 2011; Xiao-Jun et al., 2017). Experimental and clinical studies have demonstrated that ginseng and its metabolites can improve sleep quality through modulation of sleep-related neurotransmission and neuronal signaling pathways (Jingjing et al., 2020; Tie et al., 2023). However, the long cultivation cycle and limited availability of natural ginseng restrict its large-scale utilization and application.

Adventitious root cultures of ginseng (ADV) provide a plant biotechnology-based approach for the sustainable production of ginsenosides. ADV can be rapidly propagated under controlled conditions and has been reported to retain several biological activities comparable to those of natural ginseng, including cytoprotective, metabolic regulatory, and tissue repair effects (Hosakatte Niranjana et al., 2014; Lee et al., 2023; Xiaohao et al., 2021). Despite their increasing use as an alternative source of ginsenosides, whether ADV possess sleep-regulating activity and the underlying pharmacological mechanisms remain largely unknown.

Accumulating evidence indicates that sleep homeostasis is maintained through coordinated regulation of stress response processes (Palter et al., 1986; Rensing and Monnerjahn, 1996; Rosenzweig et al., 2019). Sleep deprivation induces cellular stress responses and disrupts circadian clock function, leading to impaired neuronal homeostasis and metabolic imbalance (Shaw et al., 2002; Yamazaki et al., 2024). Dysregulation of these interconnected processes contributes to sleep disturbances and neurophysiological dysfunction, suggesting that their modulation may represent an important mechanism underlying the effects of sleep-regulating agents.

*Drosophila melanogaster* is a well-established model for sleep research due to its conserved sleep-regulatory pathways and suitability for behavioral, genetic, and molecular investigations (J C et al., 2000). Therefore, the present study employed a sleep deprivation-induced *Drosophila* model to evaluate the efficacy and safety of ADV. Transcriptomic analysis was performed to investigate potential molecular mechanisms, while LC–MS/MS profiling and bioactivity-guided assays were conducted to identify active ginsenosides. This study aimed to characterize the sleep-promoting effects of ADV and to elucidate the molecular processes underlying its regulation of sleep homeostasis.

## 2 Materials and methods

### 2.1 Reagents

Purified ginsenoside standards (Rd, Rc, Rb2, Re, F2, chromatographic purity ≥98%) were purchased from Shanghai Yuanye Bio-Technology Co., Ltd. (Shanghai, China).

### 2.2 Cultivation conditions and quality control of ADV

Wild-ginseng adventitious roots (ADV, batch No. DBGS-YZ-2018005-0501) were obtained from Tonghua Herbal Biotechnology Co., Ltd. The stock culture line DBGS-WGOS-2011-1114 was initiated from surface-sterilized root explants of wild-ginseng. Intact wild ginseng roots free of wounds and insect damage were cleaned with tap water, surface-disinfected, and sliced to generate adventitious root cultures. After screening, this culture line exhibited stable subculture performance and was free of exogenous contamination. Traceable voucher reference documents for the source plant are maintained as non-public proprietary records by the manufacturer.

The wild-ginseng adventitious roots obtained from culture were rinsed with purified water to remove residual liquid medium. Surface water was removed, and materials were dried by forced–air drying at 60 °C ± 2 °C. The dried product was sealed and stored in a cool–dry place protected from direct sunlight.

Quality control specifications for dried wild-ginseng adventitious roots included moisture ≤12.0% (determined according to GB 5009.3) and total ginsenosides ≥2.50% (determined according to GB/T 19506). Batch DBGS-YZ-2018005-0501 was produced by mixing five independent dried sub-batches (DBGSYZ20180050101-DBGSYZ20180050105). The sub-batches ranged from 3.41% to 3.85% for total ginsenosides and 4.7%–5.2% for moisture, with individual dry weights between 119 g and 137 g. The final mixed batch weighed 637 g, with composite moisture of 4.9% and total ginsenosides of 3.58%, and was prepared on 01 September 2022.

The dried wild-ginseng adventitious root material was ground into fine powder and directly incorporated into *Drosophila melanogaster* diet for feeding assays. No further extraction or purification steps were applied to the ADV material.

### 2.3 *Drosophila* culture and husbandry

Canton-S (CS) wild-type *Drosophila melanogaster* were obtained from the College of Life Sciences, Northeast Normal University (Changchun, China). Flies were maintained under standard conditions (25° C, 70% relative humidity, 12 h light/dark cycle) on a wheat cream agar medium supplemented with yeast as a protein source (Yang et al., 2021). Age-synchronized 7-day-old male flies were used in all experiments.

### 2.4 Sleep-deprivation assay and sleep-behavior monitoring

Sleep deprivation was performed in accordance with a previously published mechanical-stimulation protocol (Weiss et al., 2024). As shown in Figure 1, flies received 7 consecutive days of feeding treatment with control diet, SD basal diet, or SD + ADV diet before the sleep-deprivation challenge. Subsequently, a single 12-h nocturnal sleep-deprivation session was implemented from 20:00 to 08:00 using a rotating device operating at 120 rpm. After termination of mechanical rotation, no further mechanical stimulation was administered during the following 24-h behavioral and sleep assessment period. Control flies were maintained under identical environmental conditions in the absence of mechanical sleep-deprivation stimulation. The effectiveness of sleep deprivation was evaluated after deprivation by analyzing sleep parameters acquired with the *Drosophila* Activity Monitoring (DAM) system (Thimgan et al., 2015).

**FIGURE 1:**
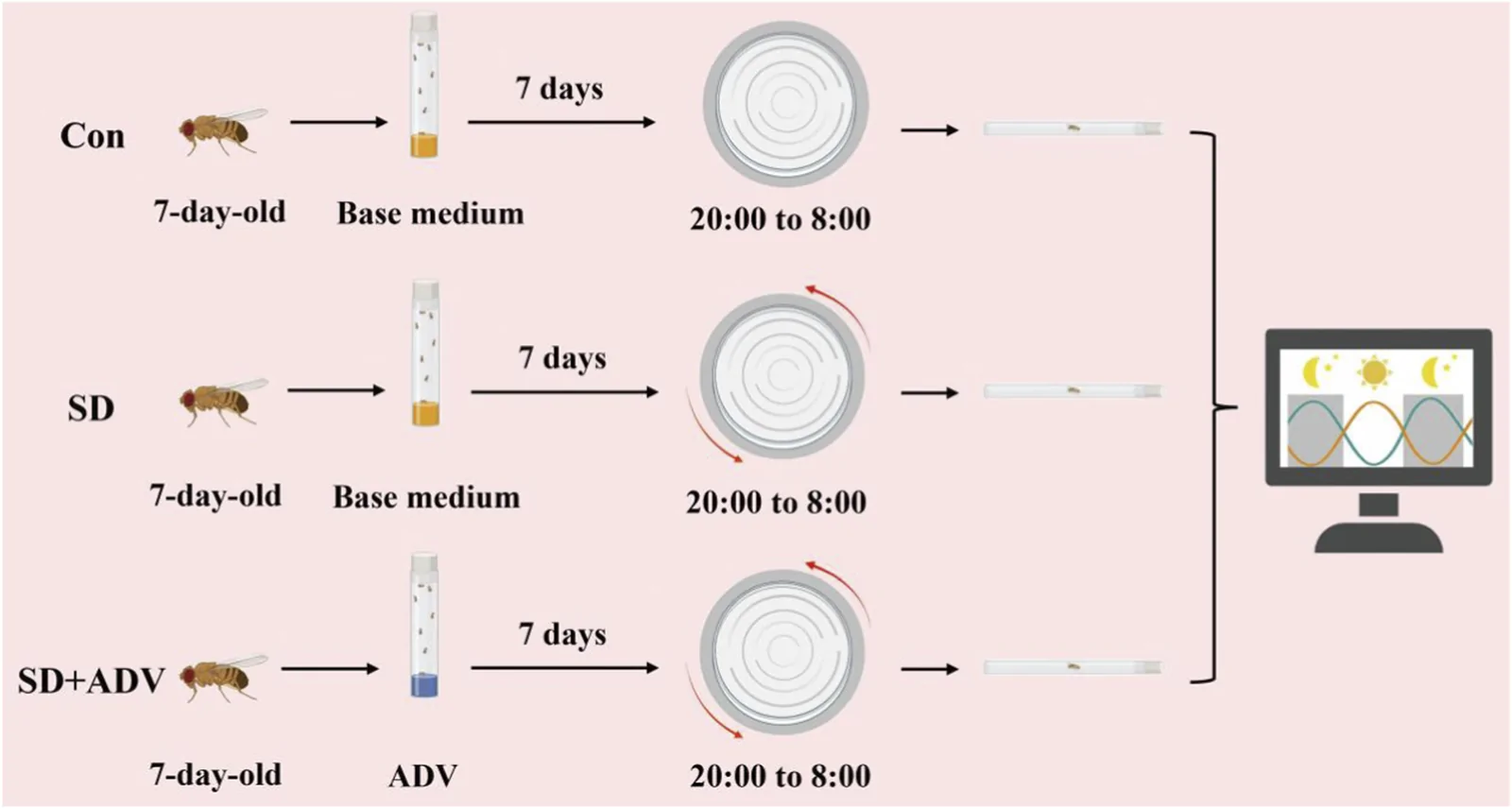
Schematic workflow for fly feeding pre-treatment, single-episode mechanical sleep-deprivation challenge, ADV intervention, and subsequent sleep-wake behavioural monitoring. Seven-day-old male Canton-S flies received 7 consecutive days of dietary pre-treatment (control diet, SD basal diet or SD + ADV diet). Afterwards flies were subjected to a single 12-h mechanical sleep-deprivation session (20:00–08:00). Mechanical rotation was stopped after deprivation, and sleep-wake activity was continuously recorded over a 24-h period by the DAM system. Con, control group; SD, sleep-deprivation group; SD + ADV, sleep-deprived flies receiving ADV-supplemented diet. Created with BioRender.com (https://BioRender.com), licensed under the BioRender Academic Publication License.

Seven-day-old male flies were randomly divided into three groups: control, sleep deprivation (SD), and SD + ADV (*n* = 32 per group). For the SD + ADV group, flies were maintained on ADV-supplemented medium throughout the 7-day pre-treatment period before the single 12-h sleep-deprivation challenge. ADV powder was incorporated into fly food at 1% (w/v), corresponding to 1 g dry ADV powder per 100 mL standard fly food. This concentration was selected based on preliminary dose-optimization experiments evaluating 0.5%, 1%, and 2% (w/v) ADV supplementation (Supplementary Figure S1). ADV-containing food was prepared by thoroughly mixing the powder into the medium to guarantee homogeneous compound exposure. According to previously reported ginsenoside dosing concentrations used in *Drosophila melanogaster* models, a monomer ginsenoside concentration of 1 mg/mL was referenced in our experimental design (Fu et al., 2022). The measured water content of this batch of ADV raw material was 4.9%, giving a fresh-to-dry conversion factor (dry weight/fresh weight) of 0.951. This factor was used to convert fresh-weight-based ginsenoside contents to mass per gram dry ADV powder, from which nominal concentrations of total ginsenosides and major individual ginsenosides in the final 1% ADV diet were calculated. The calculated nominal total ginsenoside concentration in 1% ADV food is 0.376 mg/mL. Since food intake per individual fly was not quantified in this study, only nominal concentrations within the food matrix are reported, and no per-fly dose estimates are provided.

Sleep behavior was recorded using the *Drosophila* Activity Monitoring system (DAM, Trikinetics, United States). Individual flies were placed in glass tubes (5 × 65 mm) containing basal fly food at one end and sealed with cotton plugs. Activity was recorded in 5-min bins. Sleep was defined as a period of inactivity lasting ≥5 min. The following parameters were analyzed: total sleep duration, daytime and nighttime sleep, sleep latency, sleep fragmentation, and deep sleep (defined as sleep bouts >15 min) (Bart et al., 2013).

### 2.5 Preference and addiction-related assays

A modified capillary feeder (CAFE) assay was used to evaluate feeding preference (Anita V and Ulrike, 2009). Flies were provided with liquid food containing sucrose/yeast extract with or without ADV or ethanol (15% ethanol as positive control).

Preference index (PI) was calculated as: PI = (consumption of test solution − control solution)/total consumption.

To assess aversion, quinine was added to the diet. Withdrawal assays were conducted by removing ADV or ethanol for 24 h after prolonged exposure (16 or 20 days), followed by re-exposure to evaluate relapse-like behavior.

### 2.6 Transcriptomic analysis

Fly heads were collected at 20:00 (ZT12, 12 h after lights-on) under a 12 h light/dark cycle (08:00–20:00 light period) by rapid freezing in liquid nitrogen and mechanical separation. Each biological replicate consisted of pooled heads from 100 flies. All groups were collected at the same Zeitgeber time to minimize circadian variation. Total RNA was extracted using TRIzol reagent (Invitrogen). mRNA was purified using a commercial kit (Thermo Fisher Scientific). Paired-end RNA-seq libraries were constructed with the NEBNext® Ultra™ RNA Library Prep Kit for Illumina® (ABclonal). Library preparation, sequencing, and bioinformatics analyses were performed by Applied Protein Technology (Shanghai, China). Libraries were sequenced on an Illumina NovaSeq 6,000 platform to generate 150 bp paired-end (PE150) reads.

Three biological replicates were included for each experimental group. Correlation analysis among biological replicates demonstrated high reproducibility (*R* > 0.8). Approximately 6 Gb of sequencing data were generated for each sample. Raw sequencing reads were filtered by removing low-quality reads (average base quality score <20) and reads containing excessive ambiguous bases (N > 5) to obtain clean reads. Clean reads were aligned to the *Drosophila melanogaster* reference genome GCA_029775095.1 using HISAT2. Gene-level read counts were quantified using featureCounts, and differential expression analysis was performed using the DESeq2 R package. Genes with an adjusted *p*-value (padj) < 0.05, calculated using the Benjamini–Hochberg method for false discovery rate (FDR) correction, and |log_2_ (fold change)| > 1 were considered differentially expressed genes (DEGs).

Gene Ontology (GO) enrichment analysis was performed to classify differentially expressed genes into Biological Process (BP), Molecular Function (MF), and Cellular Component (CC) categories. KEGG pathway enrichment analysis was performed based on *Drosophila melanogaster* gene annotation using KEGG Orthology (KO)-based pathway mapping. The RNA-seq datasets have been deposited in the Gene Expression Omnibus (GEO) database under accession number GSE341942.

### 2.7 Protein-protein interaction (PPI) network analysis

Differentially expressed genes were imported into the STRING database to construct protein-protein interaction networks for *Drosophila melanogaster*. Interaction pairs with a combined confidence score >700 were retained for network reconstruction.

### 2.8 Determination of ginsenosides

Total ginsenoside content of dried wild-ginseng adventitious root sample (batch DBGS-YZ-2018005-0501) was determined according to GB/T 19506 using a colorimetric method, and the result is expressed on a dry-weight basis, measured in technical duplicates. This colorimetric assay determines the total ginsenoside pool, including numerous minor ginsenoside homologues that are not individually quantified by HPLC.

17 targeted compounds in ADV were quantified by liquid chromatography–tandem mass spectrometry (LC–MS/MS). Briefly, appropriate amounts of ADV powder were extracted with 1 mL of 70% methanol, homogenized, and sonicated at 50° C for 30 min. After centrifugation at 13,000 rpm for 5 min, the supernatants were collected and filtered through a 0.45 μm membrane filter before LC–MS/MS analysis (Jae Won et al., 2017).

Chromatographic separation was performed using an Agilent 1290 UPLC system coupled with a 6470B triple quadrupole mass spectrometer (Agilent Technologies, United States) equipped with an ACQUITY UPLC BEH C18 column (2.1 × 100 mm, 1.7 μm). The column temperature was maintained at 40° C, the flow rate was set at 0.3 mL/min, and the injection volume was 5 μL. Detailed gradient conditions are provided in Table 1. The mass spectrometer was operated in negative electrospray ionization (ESI−) mode.

**TABLE 1:** Mobile phase gradient ratio for HPLC.

Time (min)	Flow rate (mL/min)	A% (0.1% methanoic acid)	B% (acetonitrile)
0.00	0.3	85	15
0.50	0.3	85	15
1.00	0.3	80	20
6.00	0.3	80	20
13.00	0.3	70	30
23.00	0.3	65	35
24.00	0.3	62	38
27.00	0.3	40	60
31.00	0.3	10	90
32.00	0.3	85	15
35.00	0.3	85	15

Individual ginsenosides were identified based on their retention times, precursor ions, and characteristic MS/MS fragmentation patterns by comparison with authentic reference standards. Quantification was performed using calibration curves established from corresponding ginsenoside standards. The contents of ginsenosides were calculated according to the following equation: W = [(C - C_0_) *V *N] / m.

Where W represents the content of target compounds in samples (mg/kg), C represents the concentration of target compounds in sample solution (mg/L), C0 represents the concentration in blank control solution (mg/L), V represents the final volume after dilution (mL), N represents the dilution factor, and m represents the sample weight (g).

This LC-MS/MS workflow was implemented as a targeted quantitative characterization for ginsenoside composition in wild ginseng adventitious root powder, rather than a fully prospectively validated regulatory-grade bioanalytical method. We reviewed available original experimental records concerning extraction recovery, LOD/LOQ, precision, repeatability, sample stability, matrix effects, and precursor-product collision-energy parameters. Analytical parameters supported by reliable primary source records have been compiled into supplementary information. Parameters that were not systematically assessed in the original experiment, and for which no raw experimental records exist, were not retrospectively generated or computationally inferred.

### 2.9 Screening of representative ginsenosides

No chromatographic fractionation of ADV was performed for ginsenoside screening. Based on the quantitative LC–MS/MS profiling of ADV, the relatively high abundance of individual ginsenosides, and previous reports describing their neuroprotective and sleep-related activities, five representative ginsenosides (Rd, Rc, Rb2, Re, and F2) were selected for biological evaluation. Purified ginsenoside standards (chromatographic purity ≥ 98%, according to the manufacturer’s certificate of analysis) were obtained from Shanghai Yuanye Bio-Technology Co., Ltd. No additional in-house purity verification or compound stability testing was performed in this study. Stock solutions for each ginsenoside were prepared in DMSO at room temperature with sonication, without heating. These DMSO stock solutions were added to molten fly food after the medium had cooled to ≤ 50° C and thoroughly mixed to achieve a final compound concentration of 1 mg/mL. The final DMSO concentration was maintained at 0.2% (v/v) across all experimental and vehicle-control groups. A DMSO-vehicle-control food containing 0.2% (v/v) DMSO served as the sole control condition; no solvent-free untreated food group was included in this set of single-ginsenoside feeding experiments. Ginsenoside-supplemented food was freshly prepared for each experiment, and fly vials were renewed every 3 days to mitigate potential compound degradation. Experimental flies were reared under the same base circadian conditions as described above. Flies received continuous ginsenoside-supplemented diet for 7 days. During this feeding period, flies were subjected to daily sleep-deprivation using the identical procedure described in the preceding sections, followed by behavioural recording. This study employed a single-point screening design at 1 mg/mL; dose-response experiments and composition-matched matrix reconstitution were not performed.

### 2.10 Statistical analysis

All statistical analyses were performed using GraphPad Prism software. Prior to formal comparison, the normality of data distribution was assessed by the Shapiro-Wilk test, and homogeneity of variance was evaluated. For datasets satisfying normality and equal-variance assumptions, parametric statistical tests were applied. When normality or variance assumptions were violated, non-parametric tests were used instead. Repeated-measures or time-course behavioural data derived from *Drosophila* activity monitoring (DAM) were analysed by repeated-measures ANOVA where appropriate. Multiplicity control was implemented for multiple-group comparisons using *post hoc* correction procedures as specified in individual figure legends. All behavioural experiments were performed using independent biological replicates; individual flies served as experimental units. Flies were randomly assigned to experimental groups during vial preparation. Blinding was performed during behavioural data acquisition and quantitative analysis. No pre-defined sample-exclusion criteria were applied in this study. Statistical significance was defined as *p* < 0.05.

## 3 Results

### 3.1 ADV improves sleep parameters in sleep-deprived *Drosophila melanogaster*

To evaluate the sleep-promoting effects of ADV, multiple sleep-related parameters were assessed in sleep-deprived *Drosophila melanogaster*. Sleep deprivation significantly reduced daytime sleep, nighttime sleep, and total sleep duration compared with the control group, whereas ADV treatment markedly increased sleep duration in sleep-deprived flies (Figures 2A–C). Sleep fragmentation was further analyzed by measuring sleep episode number and mean sleep duration per episode. No significant difference in sleep episode number was observed among groups (Figure 2D). However, mean sleep duration per episode was significantly reduced following sleep deprivation and restored by ADV treatment (Figure 2E). In addition, deep sleep duration was markedly decreased in sleep-deprived flies and significantly increased following ADV administration (Figure 2F).

**FIGURE 2:**
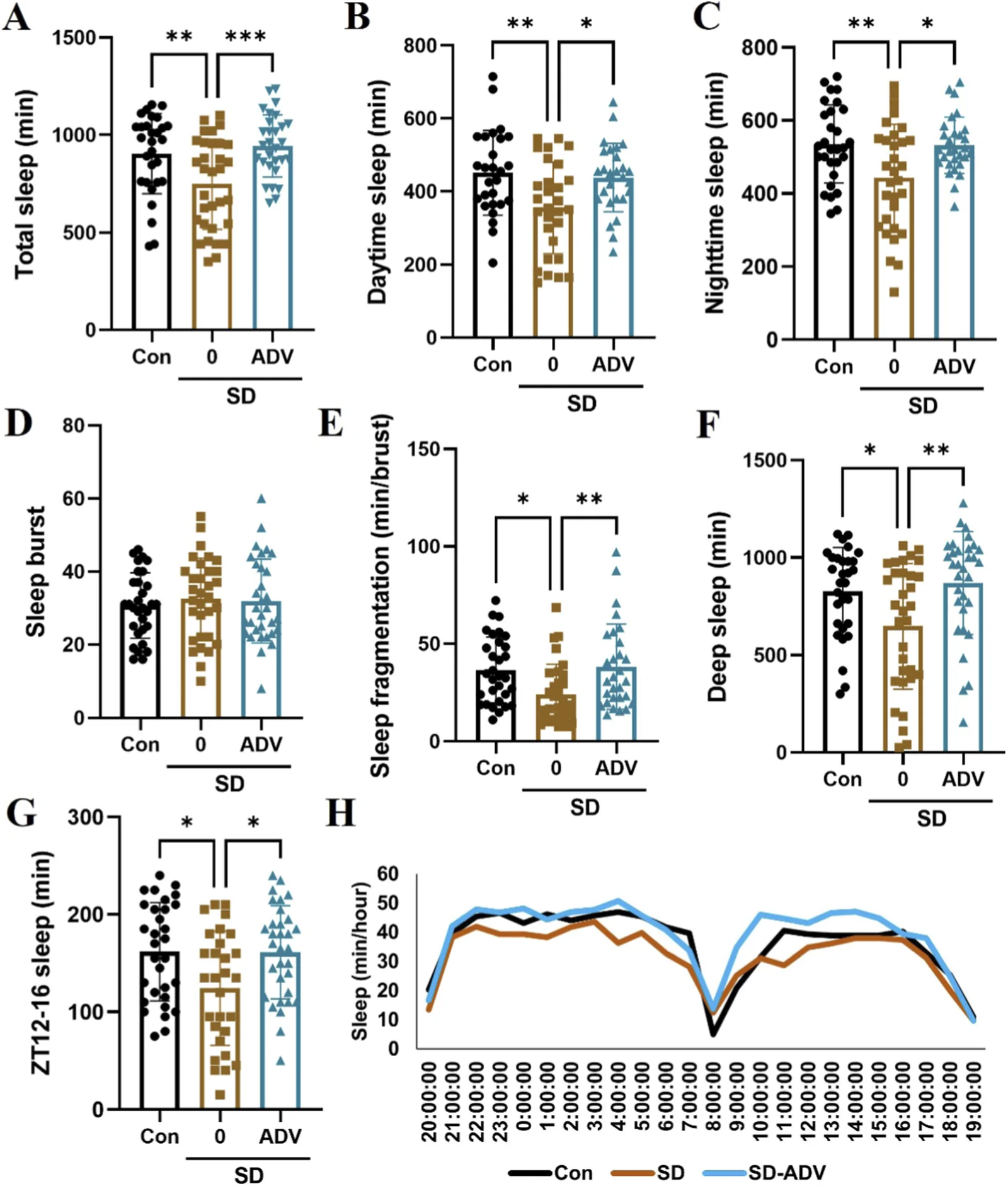
Effect of ADV on sleep parameters in sleep-deprived *Drosophila* model. **(A)** Average sleep duration length in LD conditions (12 h light, 12 h dark). **(B)** Average sleep duration length during daytime. **(C)** Average sleep duration length during nighttime. **(D)** The number of sleep burst in LD conditions. **(E)** Fragmented sleep architecture (Average bout length of sleep). **(F)** The duration of deep sleep (A sleep duration of more than 15 min). **(G)** Average sleep duration length during ZT12–16 (The first 4 h after lights-off). **(H)** Profiles of sleep rhythm. Dried ADV powder (batch DBGS-YZ-2018005-0501; residual moisture content = 4.9%, dry-matter fraction = 95.1%, fresh-to-dry conversion factor DW/FW = 0.951) was supplemented into standard fly food at 1% (w/v, dry-powder basis). The nominal total ginsenoside concentration of the 1% ADV diet was 358.0 mg/L, and the summed concentration of 17 major HPLC-quantified ginsenoside markers was 49.91 mg/L (see Supplementary Material S1 for full calculation). Flies were maintained at 25° C under a 12 h light-dark cycle. Con, non-sleep-deprived control group; 0, sleep-deprived solvent control group; ADV, sleep-deprived flies fed with ADV-supplemented diet. Data are presented as mean ± SD (n = 32 biological replicates per group). Statistical analysis was performed using one-way ANOVA followed by Tukey’s multiple comparisons test. *p < 0.05, **p < 0.01, ***p < 0.001.

To evaluate sleep latency, sleep duration during the first 4 h after lights-off (ZT12–16) was analyzed. Sleep deprivation significantly reduced ZT12–16 sleep duration, whereas ADV treatment effectively reversed this alteration (Figure 2G). Analysis of sleep rhythm further showed that sleep deprivation reduced rhythm amplitude without affecting rhythm phase, while ADV treatment partially restored rhythm amplitude (Figure 2H).

To further validate the sleep-promoting activity of ADV, six-year-old cultivated ginseng (GEN) was included as a positive control in preliminary experiments. Similar to ADV treatment, GEN significantly increased total sleep duration in sleep-deprived *Drosophila melanogaster*, and no significant difference was observed between the ADV and GEN groups (Supplementary Figure S2). These results indicate that ADV exhibits comparable sleep-promoting activity to cultivated ginseng under the present experimental conditions. Collectively, these results indicate that ADV effectively improves sleep quality and sleep stability in sleep-deprived *Drosophila melanogaster*.

### 3.2 ADV does not markedly disrupt locomotor activity patterns or circadian behavioral rhythms in sleep-deprived *Drosophila melanogaster*

To determine whether the sleep-protective effects of ADV were associated with altered locomotor behavior, locomotor activity was evaluated across experimental groups. Under sleep-deprivation conditions, we observed moderate changes in raw daytime locomotor activity in the SD + ADV group. However, no significant intergroup differences were detected for wake-normalised movement counts per minute or the duration of individual activity episodes (Figures 3D,E). Furthermore, circadian locomotor-rhythm analysis revealed no obvious changes in rhythmic profiles between groups (Figure 3F).

**FIGURE 3:**
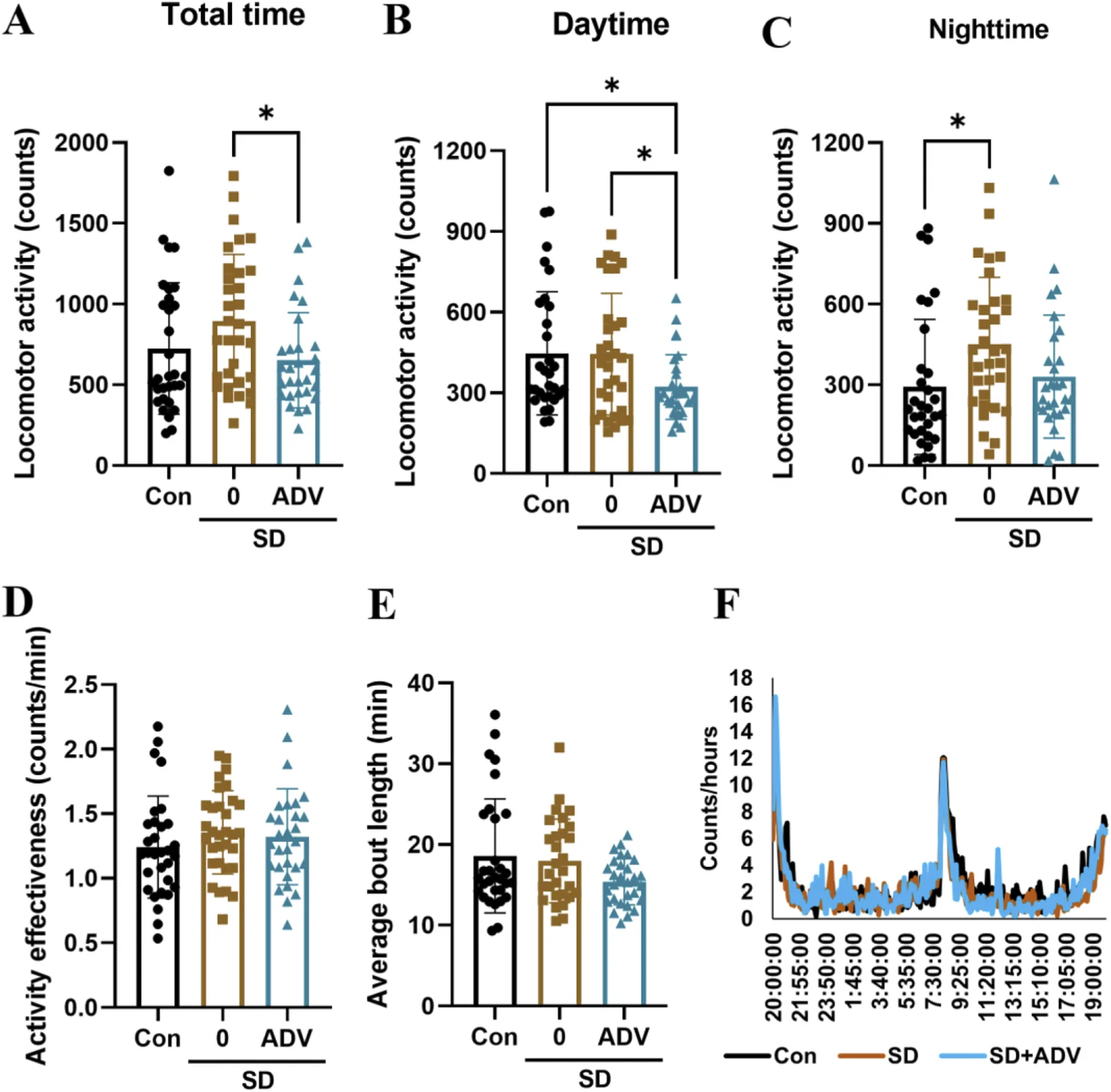
Effect of ADV on locomotor activity of sleep-deprived *Drosophila* model. **(A)** Average locomotor activity counts per day in LD conditions (12 h light, 12 h dark). **(B)** Average locomotor activity counts during daytime. **(C)** Average locomotor activity counts during nighttime. **(D)** Average locomotor activity counts per min during wakefulness **(E)** Average activity bout length (min) in light conditions. **(F)** Profiles of sleep activity. Dried ADV powder (batch DBGS-YZ-2018005-0501; residual moisture content = 4.9%, dry-matter fraction = 95.1%, fresh-to-dry conversion factor DW/FW = 0.951) was supplemented into standard fly food at 1% (w/v, dry-powder basis). The nominal total ginsenoside concentration of the 1% ADV diet was 358.0 mg/L, and the summed concentration of 17 major HPLC-quantified ginsenoside markers was 49.91 mg/L (see Supplementary Material S1 for full calculation). Flies were maintained at 25° C under a 12 h light-dark cycle. Con, non-sleep-deprived control group; 0, sleep-deprived solvent control group; ADV, sleep-deprived flies fed with ADV-supplemented diet. Data are presented as mean ± SD (n = 32 biological replicates per group). Statistical analysis was performed using one-way ANOVA followed by Tukey’s multiple comparisons test. *p < 0.05.

Important caveats apply to these interpretations. The absence of a non-sleep-deprived ADV-treated control group prevents assessment of ADV-driven changes in baseline locomotor activity. The lack of statistically significant differences in wake-normalised activity does not constitute definitive proof that ADV lacks sedative-like activity. Collectively, within the tested sleep-deprivation experimental context, these data indicate that ADV did not produce broad disruption of measured locomotor-rhythm or wake-activity parameters in sleep-deprived files.

### 3.3 ADV does not induce addiction-like behavior *in Drosophila melanogaster*

To evaluate the safety profile of ADV, addiction-related behavioral assays were performed using ethanol as a positive control. A time-dependent increase in consumption and preference index was considered indicative of addiction-like behavior. As shown in Figures 4A–C, flies exhibited a progressive increase in ethanol preference over time, whereas no significant preference for ADV was observed, suggesting that ADV did not induce addiction-like behavior.

**FIGURE 4:**
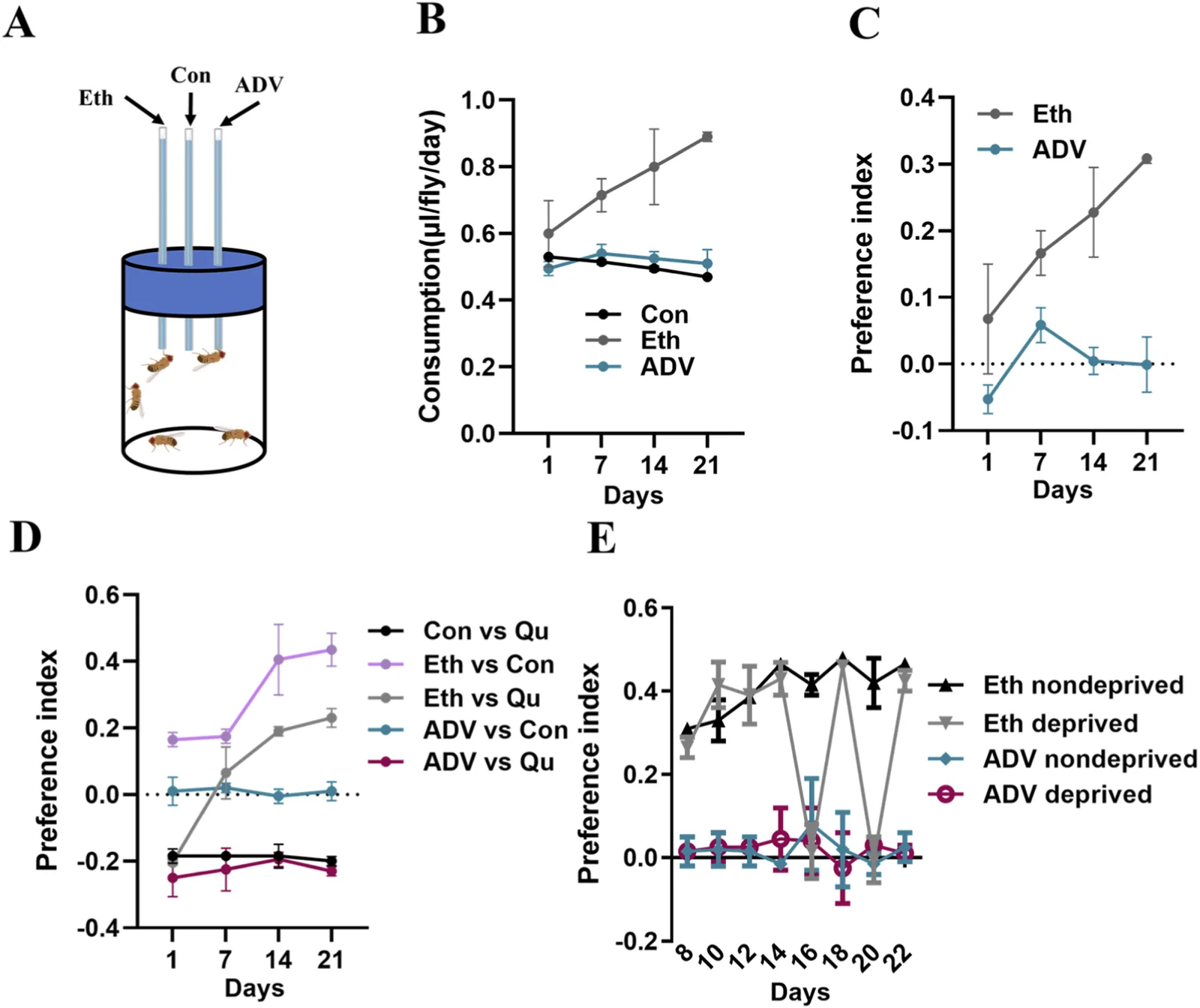
Attraction, aversion and addictive characteristics of *Drosophila melanogaster* to ADV. **(A)** Schematic of the preference assay, Flies choose among liquid food containing 0%, 15% ethanol or 1% ADV. Each food type is presented in 2 capillaries to increase the food supply and decrease variability. **(B)** Preference assay for ethanol and ADV in *Drosophila melanogaster*. **(C)** PI calculated from consumption values. **(D)** Effects of ethanol and ADV on quinine aversion in *Drosophila melanogaster*. **(E)** Analysis of addiction characteristics of *Drosophila melanogaster* to ethanol and ADV.

A quinine-based aversion assay was further conducted to evaluate compulsive preference behavior. Despite the strong aversive taste of quinine, flies progressively increased their preference for ethanol-containing food, whereas no comparable increase in preference was observed for ADV-containing food during the experimental period (Figure 4D). In addition, withdrawal and re-exposure experiments demonstrated that ethanol deprivation significantly reduced the preference index, which rapidly recovered following ethanol re-administration, indicating a withdrawal-like response. In contrast, ADV withdrawal did not produce comparable alterations in feeding preference. Collectively, these findings suggest that ADV exhibits a low risk of inducing addiction-like behavioral responses in *Drosophila melanogaster*.

### 3.4 Transcriptomic analysis of the mechanisms underlying the sleep-promoting effects of ADV

To explore the molecular mechanisms underlying the sleep-promoting effects of ADV, transcriptomic profiling of *Drosophila* heads was performed using high-throughput RNA sequencing. Differential gene expression analysis between the control and sleep deprivation groups revealed that sleep deprivation upregulated 182 genes and downregulated 72 genes in *Drosophila* heads (Figures 5A,B).

**FIGURE 5:**
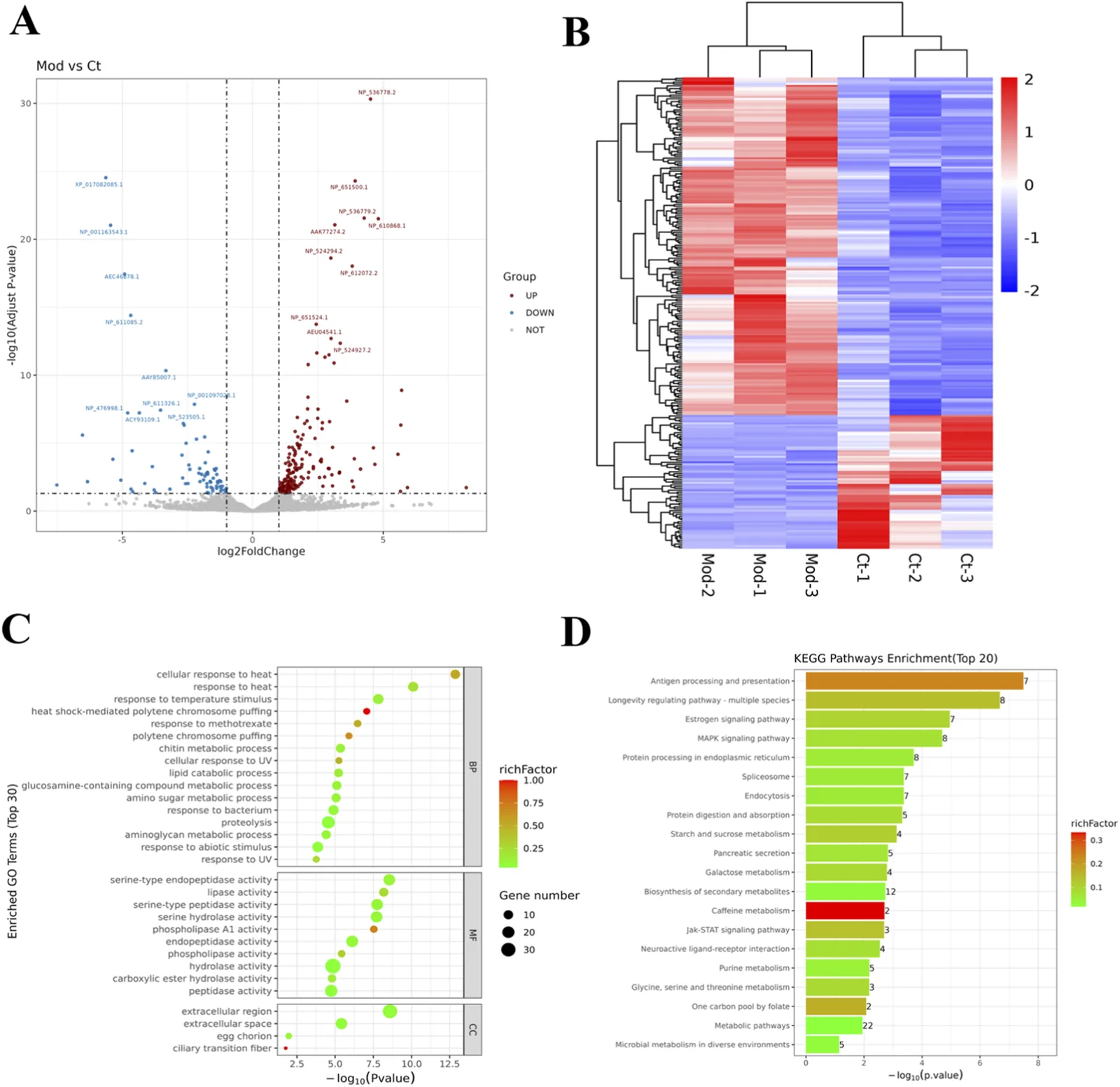
Effects of sleep deprivation on the transcriptome of *Drosophila* head tissue. **(A)** Volcano map of differential genes between sleep deprived *Drosophila* model group and control group. **(B)** Cluster analysis of differential genes between sleep deprived *Drosophila* model group and control group. **(C)** GO enrichment of differential genes between sleep deprived *Drosophila* model group and control group. Biological Process (BP), Molecular Function (MF), and Cellular Component (CC). **(D)** Enrichment of KEGG pathway of differential genes between sleep deprived *Drosophila* model group and control group. Con, control group; Mod, sleep deprivation group.

Gene Ontology (GO) enrichment analysis indicated that the differentially expressed genes were mainly associated with heat response, proteolysis, lipid metabolism, and extracellular region-related processes (Figure 5C). KEGG pathway analysis identified 58 significantly enriched pathways, including antigen processing and presentation, longevity regulating pathway, estrogen signaling pathway, and neuroactive ligand–receptor interaction (Figure 5D).

We next analyzed transcriptomic alterations between the ADV-treated and sleep deprivation groups. Compared with sleep-deprived flies, ADV treatment upregulated 168 genes and downregulated 49 genes (Figures 6A,B). GO enrichment analysis showed that the altered genes were primarily associated with heat response, proteolysis, hypoxia response, and sterol homeostasis, whereas KEGG analysis revealed enrichment in pathways related to estrogen signaling, longevity regulation, MAPK signaling, and neuroactive ligand–receptor interaction (Figures 6C,D).

**FIGURE 6:**
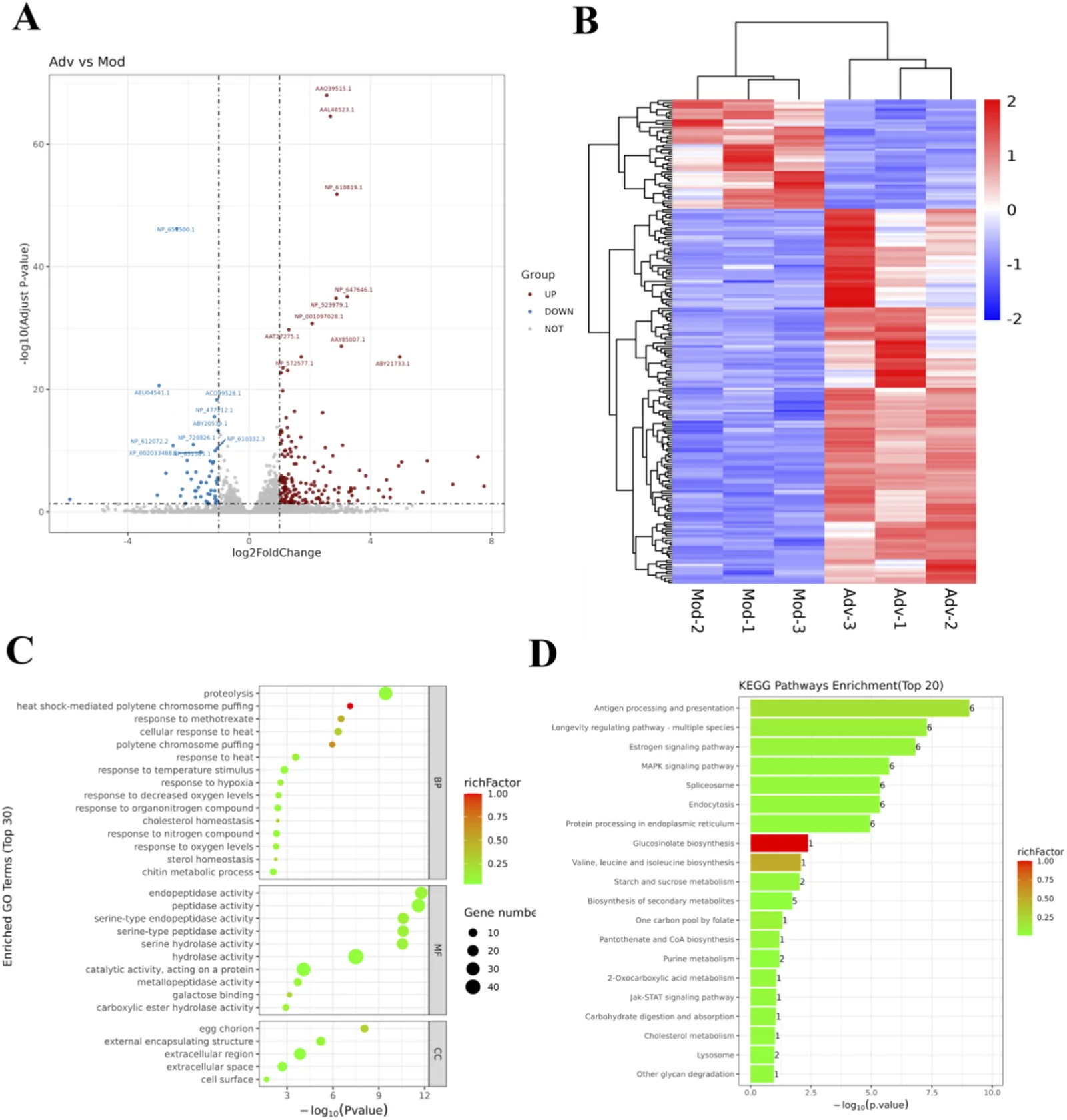
Effects of ADV on the transcriptome of head tissue in sleep deprived *Drosophila* model. **(A)** Volcano map of differential genes between sleep deprived *Drosophila* model group and ADV treated group. **(B)** Cluster analysis of differential genes between sleep deprived *Drosophila* model group and ADV treated group. **(C)** GO enrichment of differential genes between sleep deprived *Drosophila* model group and ADV treated group. Biological Process (BP), Molecular Function (MF), and Cellular Component (CC). **(D)** Enrichment of KEGG pathway of differential genes between sleep deprived *Drosophila* model group and ADV treated group. Mod, sleep deprivation group; ADV, sleep deprivation + ADV treatment group.

Further comparative analysis revealed that 39 genes were significantly upregulated following sleep deprivation and partially restored toward control levels after ADV treatment. These genes were mainly associated with the heat shock response, including *Hsp68*, *Hsp70Aa*, *Hsp70Ba*, *Hsp70Bb*, and *Hsp70Bbb*, as well as cytochrome P450-related genes and the nuclear transcription factor Relish (Figures 7A,B). In contrast, 33 genes that were downregulated by sleep deprivation showed recovery trends following ADV treatment.

**FIGURE 7:**
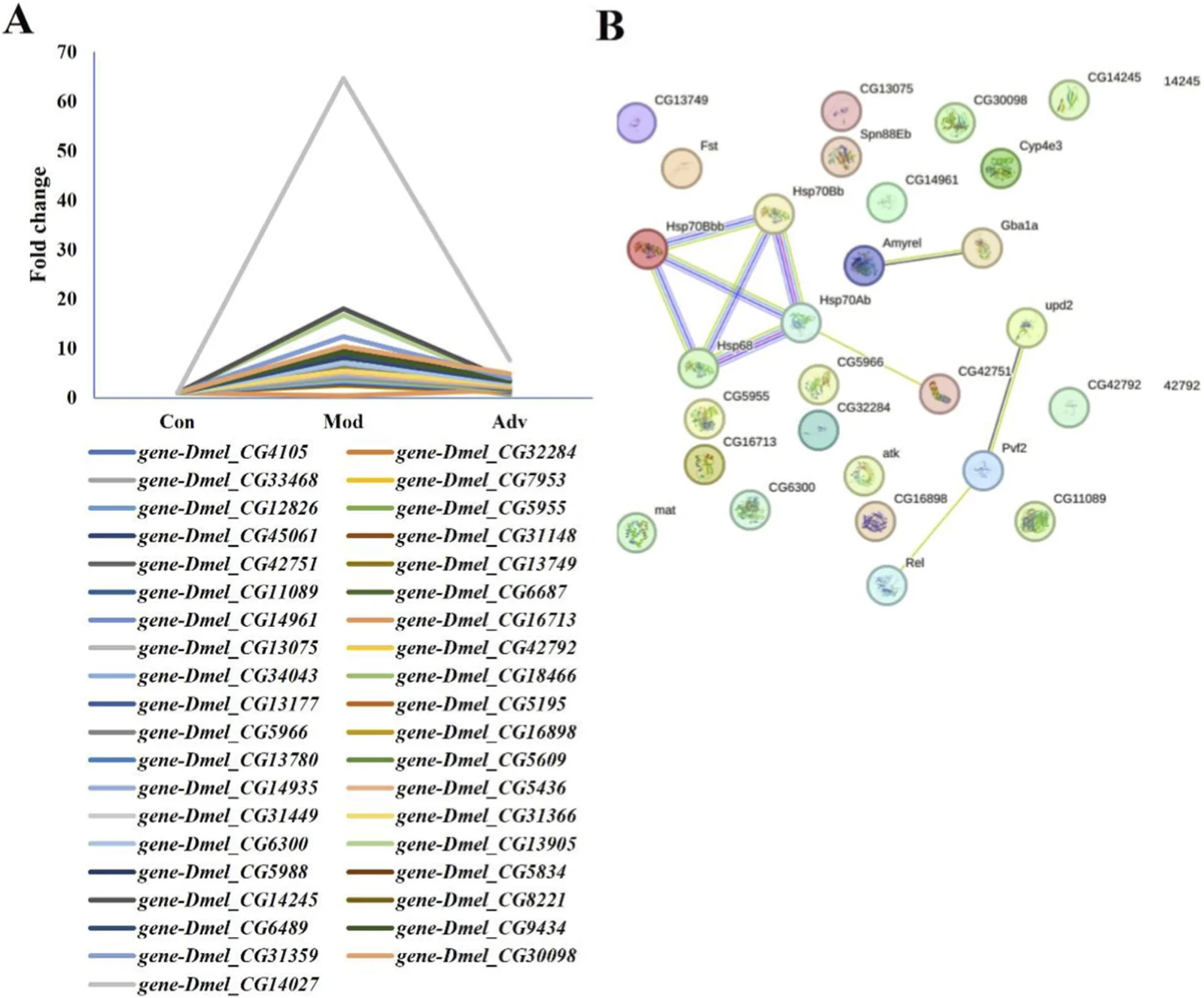
Genes in head tissue of a sleep-deprived *Drosophila* model with ADV reversing expression trends. **(A)** Genes that were highly expressed and inhibited by ADV in sleep-deprived *Drosophila* models. **(B)** PPI of the genes that were highly expressed and inhibited by ADV in sleep-deprived *Drosophila* models. Con, control group; Mod, sleep deprivation group; ADV, sleep deprivation + ADV treatment group.

### 3.5 Composition of ginsenosides in ADV and comparison of the sleep-regulating effects of major monomers

The ginsenoside composition of ADV was characterized by LC-MS/MS. As shown in Table 2 and Figure 8A, a total of 17 ginsenosides were identified, among which Rd, Rc, Rb2, Re, and F2 were the major metabolites. To further investigate whether these abundant ginsenosides contributed to the sleep-regulating effects of ADV, representative ginsenoside monomers were selected based on their abundance in ADV and previously reported neuroprotective activities, and their effects on sleep-related parameters were evaluated. As shown in Figures 8B–G, the tested ginsenosides exhibited differential effects on sleep behavior. Among them, ginsenoside Rc showed the strongest sleep-promoting activity, followed by F2, as indicated by increased total sleep duration, enhanced sleep during ZT0–4, and reduced sleep fragmentation. In contrast, ginsenosides Rd, Rb2, and Re showed relatively limited effects, with no significant improvement observed in the analyzed sleep parameters.

**TABLE 2:** Ginsenoside composition in ADV.

Ginsenoside monomer	Content (mg/kg FW)	Content (mg/kg DW)
Rg1	254.36 ± 5.97	267.47 ± 6.28
Re	506.47 ± 41.14	532.57 ± 43.26
Rf	33.25 ± 3.32	34.97 ± 3.49
R0	388.22 ± 12.50	408.22 ± 13.14
Rc	827.94 ± 24.60	870.60 ± 25.87
Rb2	822.14 ± 62.00	864.50 ± 65.19
Rh1	1.49 ± 0.17	1.56 ± 0.18
F1	1.26 ± 0.12	1.32 ± 0.13
Rb3	226.60 ± 14.29	238.27 ± 15.03
Rd	1286.00 ± 65.57	1352.26 ± 68.94
F2	327.16 ± 29.97	344.02 ± 31.52
Rg3	32.58 ± 3.34	34.26 ± 3.51
PPT	31.27 ± 1.07	32.88 ± 1.13
CK	2.97 ± 0.05	3.12 ± 0.06
Rh2	2.46 ± 0.22	2.58 ± 0.23
Rk1	1.50 ± 0.12	1.58 ± 0.13
Rg5	1.03 ± 0.07	1.08 ± 0.07

Concentrations of individual ginsenosides are shown on a fresh-weight basis (n = 3 analytical replicates). The 17 listed ginsenosides represent only a subset of total ginsenoside-type saponins present in ADV. The sum of these 17 analytes does not equal the total ginsenoside content. All data were obtained from one single batch of ADV, material used for all *Drosophila* feeding assays.

Ginsenoside concentrations on a dry-weight basis were converted from fresh-weight data using a fresh-to-dry conversion factor (DW/FW = 0.951), corresponding to a measured water content of 4.9% for ADV raw material. Units: mg/kg dry weight of adventitious root powder.

**FIGURE 8:**
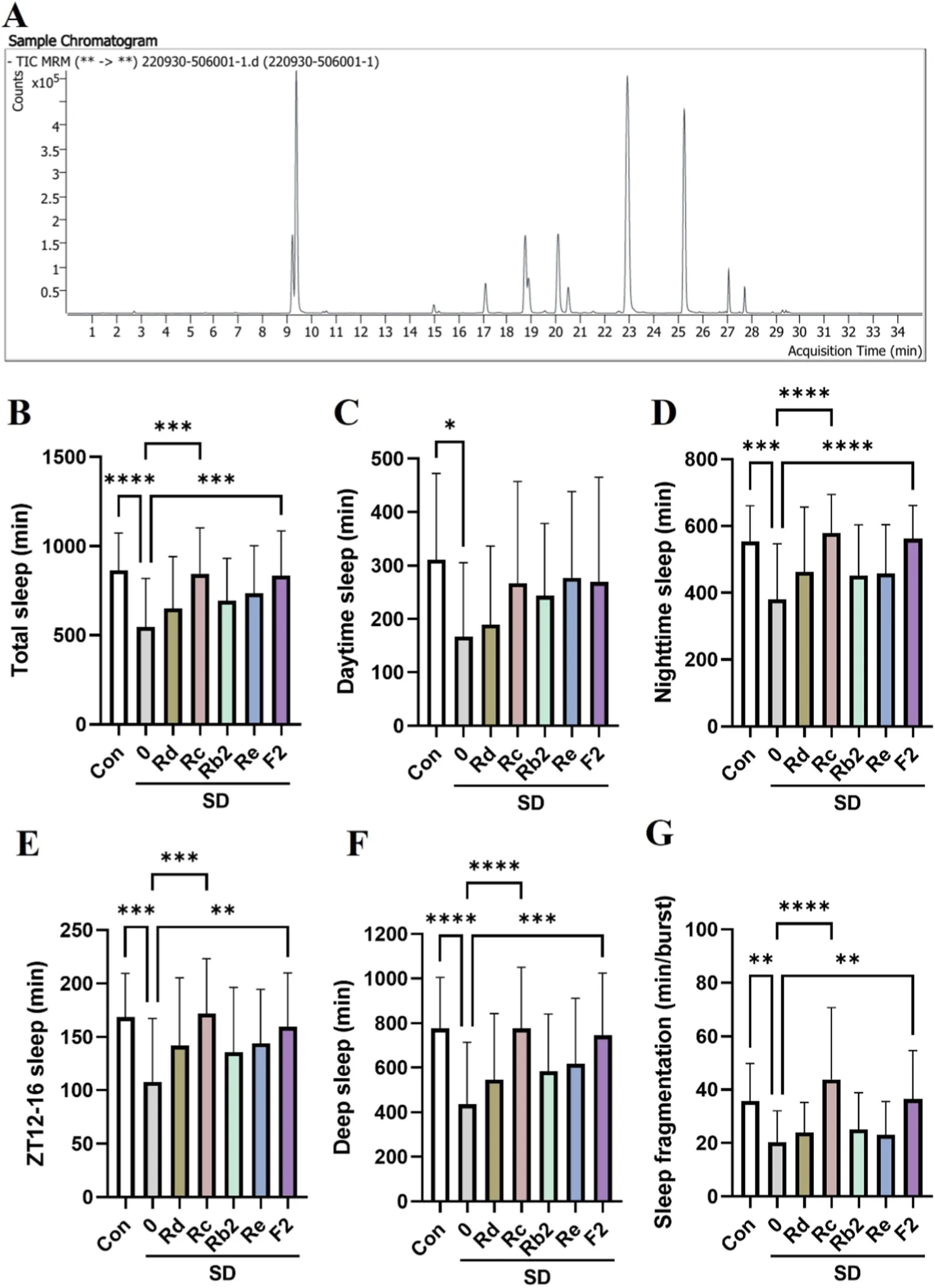
Composition of ginsenoside monomer in ADV and evaluation of sleep improvement effect. **(A)** Composition of ginsenoside monomer in ADV. **(B)**Average sleep duration length in LD conditions (12 h light, 12 h dark). **(C)** Average sleep duration length during daytime. **(D)** Average sleep duration length during nighttime. **(E)** Average sleep duration length during ZT12–16 **(F)** The duration of deep sleep (A sleep duration of more than 15 min). **(G)** Fragmented sleep architecture. Data are presented as mean ± SD (n = 32 biological replicates per group). Con: non-sleep-deprived control; 0: sleep-deprived solvent control; Rd/Rc/Rb2/Re/F2: sleep-deprived flies treated with corresponding monomer ginsenoside. Statistical analysis was performed using one-way ANOVA followed by Tukey’s multiple comparisons test. *p < 0.05, **p < 0.01, ***p < 0.001, ****p < 0.0001.

## 4 Discussion

Sleep deprivation is increasingly recognized as a major public health concern associated with cognitive dysfunction, emotional disturbance, and metabolic imbalance. Therefore, the identification of safe and effective natural products for sleep regulation has attracted growing attention (Ze-Yu et al., 2024). In the present study, wild ginseng adventitious roots (ADV) significantly improved multiple sleep-related parameters in sleep-deprived *Drosophila melanogaster*, including total sleep duration, deep sleep, sleep latency, and sleep fragmentation. Importantly, ADV did not induce addiction-like behaviors, suggesting a favorable safety profile for potential pharmacological application. The moderate reduction in daytime locomotor activity following ADV treatment may be partially attributed to enhanced sleep pressure and recovery sleep during the light phase, as ADV increased daytime sleep duration in sleep-deprived flies. Nevertheless, a mild sedative-like contribution cannot be completely excluded, and further behavioral assays are required to distinguish sleep-promoting effects from nonspecific reductions in activity. These findings demonstrate that ADV possesses sleep-promoting activity and support its potential as a sustainable source of bioactive ginsenosides.

To further elucidate the molecular basis underlying these effects, RNA-seq transcriptomic analysis was performed to characterize global gene expression changes in the head. Sleep deprivation markedly induced the expression of multiple heat shock proteins, particularly members of the Hsp70 family, which function as molecular chaperones involved in protein folding and cellular stress responses (Cirelli and Tononi, 2000; Maureen E, 2013). Increased expression of heat shock proteins is widely recognized as an indicator of proteotoxic stress under sleep-deficient conditions (San Gil et al., 2017). In the present study, ADV treatment attenuated the upregulation of *Hsp68* and *Hsp70*, suggesting that ADV may be associated with reduced transcriptional activation of stress-related responses under sleep deprivation conditions.

Although ADV treatment reversed multiple transcriptional alterations induced by sleep deprivation, we acknowledge that these changes may not exclusively represent sleep-specific homeostatic rescue and could partially reflect general stress adaptation responses to mechanical stimulation. RNA-seq transcriptomic analysis revealed that sleep deprivation affected diverse biological processes, including proteolysis, lipid metabolism, extracellular region-related processes, antigen processing and presentation, longevity regulation, estrogen signaling, and neuroactive ligand–receptor interaction. In the present study, we focused on the heat shock response because this process has been extensively implicated in sleep regulation and neuronal homeostasis. However, the current experimental design does not allow us to completely distinguish stress-buffering effects from sleep-specific regulatory mechanisms. In particular, the absence of a non-sleep-deprived ADV treatment group (Con + ADV) limits the ability to determine whether some transcriptional changes induced by ADV represent baseline regulatory effects rather than specific rescue effects under sleep-deprivation conditions. Importantly, this missing control also constrains behavioural interpretation; accordingly, we refrain from claiming that ADV specifically or causally rescues sleep loss triggered by sleep deprivation, and we cannot fully rule out the possibility of baseline locomotor suppression or a mild sedative-like effect induced by ADV. Therefore, the identified biological processes should be interpreted as potentially coordinated regulatory responses rather than a definitive signaling axis. Moreover, the present study lacks protein-level validation of key molecules involved in stress response, autophagy, and circadian regulation. Future studies incorporating Con + ADV controls, protein expression analyses, and functional intervention experiments will be necessary to further clarify and validate the broader molecular mechanisms underlying ADV-mediated sleep regulation.

Chemical profiling further demonstrated that ADV contains abundant ginsenosides, among which Rc and F2 exhibited relatively stronger sleep-promoting effects in behavioural assays. Previous studies have shown that these ginsenosides possess neuroprotective, anti-inflammatory, and metabolic regulatory activities, which may contribute to sleep regulation (Chen et al., 2025; Miao et al., 2022). These findings suggest that ginsenosides Rc and F2 may serve as candidate compounds with sleep-related activity under the tested experimental conditions. It should also be noted that our LC-MS/MS measurement represents targeted compositional quantification of ginsenosides in ADV raw material, rather than fully validated regulatory-standard bioanalytical work. Complete bioanalytical method validation was not performed in the original study and several analytical-performance parameters are unavailable, as detailed in the Methods section. Although the ginsenoside concentration data offer useful compositional information for the starting material, these quantitative outputs need to be interpreted with awareness of these analytical constraints.

Nevertheless, several caveats should be noted when interpreting these monomer-ginsenoside screening results. Individual ginsenoside standards were formulated following the identical preparation strategy used for the corresponding *Drosophila* feeding assays, with full experimental workflows documented in the Methods section. The present bioactivity evaluation was designed as a single-concentration screening assay at 1 mg/mL, and we did not perform dedicated stability assessments of each purified ginsenoside within fly food. Although ginsenoside-supplemented diet was freshly prepared and vials were renewed every 3 days to mitigate compound degradation, we cannot draw conclusions regarding long-term chemical stability of these monomers or the actual ingested exposure by individual flies. Furthermore, this screening did not include dose-response testing or matrix-reconstitution experiments, so we are unable to exclude potential synergistic or antagonistic interactions among multiple ginsenosides present in crude ADV material. Accordingly, Rc and F2 are considered candidate sleep-modulating compounds under our tested experimental conditions rather than definitively confirmed sole active constituents of ADV.

Although individual housing is required for DAM-based sleep monitoring, we acknowledge that social isolation may influence certain behavioral parameters in *Drosophila*. Since all experimental groups were maintained under identical conditions, the relative comparisons remain valid; however, future studies incorporating social environment-controlled paradigms are warranted to further evaluate the influence of social context on ADV-mediated sleep regulation. Another limitation of this study is the lack of RT-qPCR validation for individual candidate genes from transcriptomic analysis. Consequently, we cannot draw definitive conclusions regarding the expression alteration and functional significance of these single genes. Future studies are needed to verify these findings experimentally. Taken together, the present study observes associations between ADV exposure and improved sleep-related behavioural outcomes under sleep-deprivation conditions in *Drosophila melanogaster,* alongside detectable alterations in stress-response-associated biological processes. While these observations support ADV as a sustainable source of bioactive ginsenosides, the experimental setup does not allow us to confirm sleep-specific or causal mechanisms underlying these phenotypic changes, and further validation in mammalian models will be required to evaluate translational relevance.

In summary, exposure to ginseng adventitious roots correlates with improved sleep-related behaviours in the *Drosophila melanogaster* sleep-deprivation model. Under the tested experimental conditions, wake-normalised movement measures were not significantly altered in sleep-deprived flies receiving ADV treatment. These behavioural changes occur in parallel with transcriptional alterations in stress-response-related processes. Under our screening conditions, ginsenosides Rc and F2 displayed comparatively stronger sleep-related bioactivity and may represent candidate metabolites that could contribute to the observed phenotype.

## Data Availability

The datasets presented in this study can be found in online repositories. The names of the repository/repositories and accession number(s) can be found in the article/Supplementary Material.
