# Supplementary material for: Wild ginseng adventitious roots restore sleep homeostasis in sleep-deprived Drosophila melanogaster
Source: Front Pharmacol. 2026 Oct 2;17:1907250. doi: 10.3389/fphar.2026.1907250 (PMC13672481; doi:10.3389/fphar.2026.1907250)
Supplement: Supplementary file 1 [file DataSheet1.docx]

**Supplementary Table. S1 Test Results of 5 Batches of Samples**

| Sampling Point No. | Moisture Content (%) | Total Ginsenosides Content (%) |
| --- | --- | --- |
| 1 | 5.1 | 3.85 |
| 2 | 4.9 | 3.58 |
| 3 | 4.7 | 3.62 |
| 4 | 5.2 | 3.44 |
| 5 | 4.7 | 3.41 |
| RSD (%) | 4.6 | 4.9 |

**Supplementary Table. S2 Test Results of Samples from 5 Sampling Points**

| Sampling Point No. | Moisture Content (%) | Total Ginsenosides Content (%) |
| --- | --- | --- |
| 1 | 5.1 | 3.67 |
| 2 | 4.9 | 3.58 |
| 3 | 4.9 | 3.75 |
| 4 | 5.0 | 3.54 |
| 5 | 4.6 | 3.36 |
| RSD (%) | 3.8 | 4.1 |

**Supplementary Fig. S1. Effects of varying ADV concentrations on total sleep time of sleep-deprived *Drosophila melanogaster*.**

This dose‑screening experiment was performed to identify the optimal ADV concentration for subsequent bioactivity evaluation. ADV powder (batch DBGS‑YZ‑2018005‑0501; measured moisture content = 4.9 %, dry‑matter fraction = 95.1 %, fresh‑to‑dry conversion factor DW/FW = 0.951) was incorporated into standard fly food to achieve final concentrations of 0.5 %, 1 %, and 2 % (w/v, dry‑powder basis). Corresponding theoretical nominal ginsenoside concentrations in the diet were 179.0 mg/L, 358.0 mg/L, and 716.0 mg/L for total ginsenosides; summed concentrations of the 17 major HPLC‑quantified ginsenoside markers were 24.96 mg/L, 49.91 mg/L, and 99.82 mg/L, respectively (detailed calculation: Supplementary Information Text S1).

Flies were maintained at 25 °C under a 12 h light‑dark cycle. Experimental endpoint: total sleep duration (minutes). Groups: Con (control, normal sleep), SD (sleep deprivation, 0% ADV), SD+0.5% ADV, SD+1% ADV, SD+2% ADV. Statistical analysis was conducted using one‑way ANOVA followed by Tukey’s multiple‑comparison post‑hoc test. Data are presented as mean ± SD (n=32). ****p<0.0001.

**Supplementary Fig. S2** **Comparison of the sleep-promoting effects of ginseng adventitious roots (ADV) and cultivated ginseng (GEN) in sleep-deprived Drosophila melanogaster.**

ADV powder (batch DBGS‑YZ‑2018005‑0501; measured moisture content = 4.9 %, dry‑matter fraction = 95.1 %, fresh‑to‑dry conversion factor DW/FW = 0.951) was supplemented into standard fly food at 1% (w/v, dry‑powder basis). The nominal total ginsenoside concentration of the 1% ADV diet was 358.0 mg/L, and the summed concentration of 17 major HPLC‑quantified ginsenoside markers was 49.91 mg/L (detailed calculation: Supplementary Information Text S1). Flies were maintained at 25 °C under a 12 h light‑dark cycle. The experimental endpoint was total sleep duration (minutes). Experimental groups: Con (normal control), SD (sleep deprivation, vehicle control), SD+1% ADV, SD+GEN. Statistical analysis was performed using one‑way ANOVA followed by Tukey’s multiple‑comparison post‑hoc test. Data are presented as mean ± SD (n=32). *p<0.05, **p<0.01, ***p<0.001.

**Supplementary Information S1**

Sample 506001 corresponds to the ginseng adventitious root (ADV) sample used in this study.

(1) Representative LC-MS/MS MRM chromatogram and quantitative results of ginsenosides in ginseng adventitious root (ADV) sample 506001

(2) Representative standard calibration curves of selected ginsenoside monomers for LC-MS/MS MRM quantitative analysis
